# Supplementary material for: Characterization of Two-Component System CitB Family in Salmonella Pullorum
Source: Int J Mol Sci. 2022 Sep 5;23(17):10201. doi: 10.3390/ijms231710201 (PMC9456408; doi:10.3390/ijms231710201)
Supplement: Supplementary file 1 [file ijms-23-10201-s001.zip › ijms-1881983-supplementary.pdf]

Characterization of Two-Component System CitB Family in *Salmonella* Pullorum

Xiamei Kang [1#], Xiao Zhou [1#], Yanting Tang [1#], Zhijie Jiang [1], Jiaqi Chen [1], Muhammad Mohsin [1], Min Yue [1, 2, 3*]

[1] Institute of Preventive Veterinary Sciences & Department of Veterinary Medicine, Zhejiang University College of Animal Sciences, Hangzhou 310058, China
[2] Hainan Institute of Zhejiang University, Sanya, China
3 Zhejiang Provincial Key Laboratory of Preventive Veterinary Medicine, Hangzhou 310058, China
* Correspondence: Min Yue (myue@zju.edu.cn); Tel./Fax: +86-571-88982832
\# These authors contributed equally to this work.

Supplementary Figures

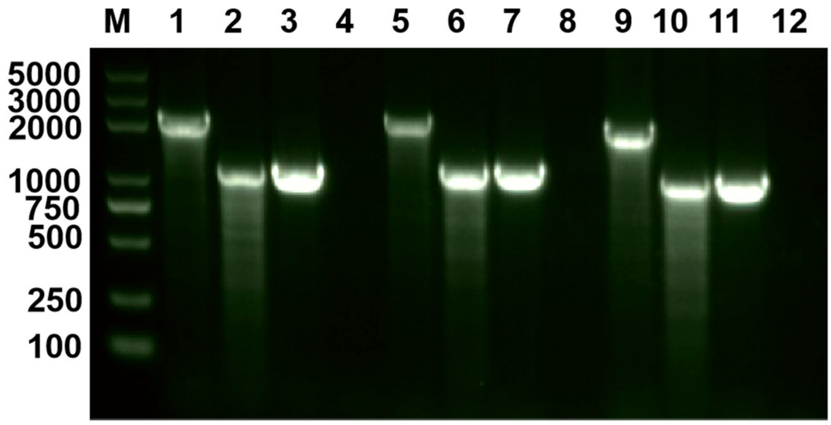

Figure S1. Identification of mutants (Δ*cit*, Δ*dcu*, Δ*dpi* and Δ3) by PCR. M: DL 5,000; 4,8,12: blank control; 1,5,9: WT; 2: Δ*cit*; 6: Δ*dcu*; 10: Δ*dpi*; 3,7,11: Δ3. 1-4 used primers which identified whether *cit* was deleted. 5-8 used primers which identified whether *dcu* was deleted. 9-12 used primers which identified whether *dpi* was deleted.

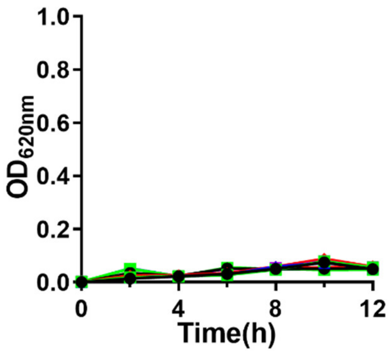

Figure S2. The growth curves of five strains in static M9 minimal medium under aerobic and anaerobic conditions, of which glucose was replaced with an equal concentration of citrate (citrate is the only carbon source).

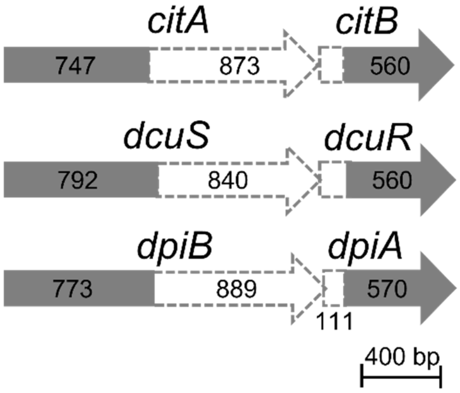

Figure S3. The deleted regions for three pairs of two component systems in wild-type strain R51. The dashed unfilled boxes indicate the deleted parts.

Supplementary Tables

Table S1. Bacterial strains and plasmids used in this study

Strains or plasmids	Genotype or relevant phenotype	Source or reference
S. Pullorum WT	Wild-type R51	Clinical isolate
Δ*cit*	WT derivative; *citAB*-knockout mutant	This study
Δ*dcu*	WT derivative; *dcuSR*-knockout mutant	This study
Δ*dpi*	WT derivative; *dpiBA*-knockout mutant	This study
Δ3	WT derivative; Δ*citAB*Δ*dcuSR*Δ*dpiBA* -knockout mutant;	This study
Escherichia coli DH5α	F^- *endA1 glnV44 thi-1 recA1 relA1 gyrA96 deoR nupG* ϕ80d*lacZ*ΔM15 Δ(*lacZYA-argF*) *U169 hsdR17* (r_K^- m_K^+) λ^-	This study
Escherichia coli TG1	[F′ *traD36 proAB lacIqZ*ΔM15] *supE thi-1* Δ(*lac-proAB*) Δ(*mcrB-hsdSM*)5(r_K^-*m_k^-*)	This study
Escherichia coli 25922	CLSI control strain for antimicrobial susceptibility testing	Lab collection
Pseudomonas aeruginosa 27853	CLSI control strain for antimicrobial susceptibility testing	Lab collection
Rosetta (DE3)	BL21 derivatives; F-*ompT hsdSB* (*rB^-mB^- gal dcm* (DE3) pRARE (*argU, argW, ilex, glyT, leuW, proL*) (CHL^R)	This study
pTarget F	Constitutive expression of sgRNA without donor editing template DNA; $SPEC^R$	[28]
pCas	Temperature-sensitive vector; Constitutive expression of cas9 and inducible expression of λ-Red and sgR; KAN^R	[28]
pET30a	Prokaryotic expression vector; KAN^R	Lab collection

CHL, chloramphenicol; SPEC, spectinomycin; KAN, kanamycin; CLSI, Clinical and Laboratory Standards Institute

Table S2. Primers used in this study

Primer's name	Sequence (5′ to 3′)
Primers for constrcution of gene deletion mutants	
cit-sgRNA-F	GTCCTAGGTATAATACTAGTAAGCTAATAACCCAGCCCTGGTTTTAGAGCTAGAAATAGCAAGTT
dcu-sgRNA-F	GTCCTAGGTATAATACTAGTTGACGATGATCTCTTCATCGGTTTTAGAGCTAGAAATAGCAAGTT
dpi-sgRNA-F	GTCCTAGGTATAATACTAGTCTGGAAGACAATGATCCTTGGTTTTAGAGCTAGAAATAGCAAGTT
sgRNA-R	AAATAGTCGGTGGTGATAAACTTAT
sgRNA vector-F	ACTAGTATTATACCTAGGACTGAGC
sgRNA vector-R	ATAAGTTTATCACCACCGACTATTT
Identify *cit*-sgRNA-F	AAGCTAATAACCCAGCCCTG
Identify *dcu*-sgRNA-F	TGACGATGATCTCTTCATCG
Identify *dpi*-sgRNA-F	CTGGAAGACAATGATCCTTG
Identify sgRNA-F	CACCGCTGATGACATCAGT
cit-Donor DNA-Xf-F	TTTTGAATTCTCTAGAGTCGTTGACCCAATGCGCTCGTTTTCGGA
cit-Donor DNA-Xf-R	TTGATAGAGGCGTATTTGCTAGCCGTTTGGTTAATGGCGG
cit-Donor DNA-Xr-F	CCGCCATTAACCAAACGGCTAGCAAATACGCCTCTATCAA
cit-Donor DNA-Xr-R	AGATCTAAGCTTCTGCAGGTTTCGAGAAAGTTATTTTTCACGCCT
dcu-Donor DNA-Xf-F	TTTTGAATTCTCTAGAGTCGTTGTGGTGACCAATATGCAGGGCAT
dcu-Donor DNA-Xf-R	CTGCATATAGATATCCAGCAGGCGTCATCCTGCGTTTTTC
dcu-Donor DNA-Xr-F	GAAAAACGCAGGATGACGCCTGCTGGATATCTATATGCAG
dcu-Donor DNA-Xr-R	TAAGCTTCTGCAGGTCGGCCTGCCGGTCACGCCGTAATGA
dpi-Donor DNA-Xf-F	TTTTGAATTCTCTAGAGTCGGGGACGGATTTTGACTATGTGGTGA
dpi-Donor DNA-Xf-R	ATTCTCGCCTGCGCCAGATTGGCAGCCCCAACATTTTTCT
dpi-Donor DNA-Xr-F	AGAAAAATGTTGGGGCTGCCAATCTGGCGCAGGCGAGAAT
dpi-Donor DNA-Xr-R	AGATCTAAGCTTCTGCAGGTCGACTCGCGCAATATTCCAGGTAGC
Donor DNA vector-F	CGACTCTAGAGAATTCAAAAAAAGC

Donor DNA-R	CACCGCTGATGACATCAGT
pCas-F	TTTGTGGAGCAGCATAAGC
pCas-R	CCGCTCATGTCTAGATTAAGA
Identify Δ*cit*-F	AAGAACGCCTGCATTATCAG
Identify Δ*cit*-R	ATAGAGTCTCTCCGGCCT
Identify Δ*dcu*-F	GGTCCATCTGATCTATTTTTCG
Identify Δ*dcu*-R	CAGTAAAGAGTAATGTTCCG
Identify Δ*dpi*-F	CATGTTCGTGATATGGCGATGAATC
Identify Δ*dpi*-R	CAGTAAAGAGTAATGTTCCG
Primers for expression of recombinant fimbrial proteins	
pET30a-F	ATGATGATGATGATGGTGCATATG
pET30a-R	GAATTCGAGCTCCGTCGACAAGC
bcfA segment-F	ACATATGCACCATCATCATCATCATGATACTACAACCGTCACTGG
bcfA segment-R	AAGCTTGTCGACGGAGCTCGAATTCTCAGGAATAAACCATGCTAA
fimA segment-F	ACATATGCACCATCATCATCATCATGCTGATCCTACTCCGGTGAGC
fimA segment-R	AAGCTTGTCGACGGAGCTCGAATTCTTATTCGTATTTCATGATAAA
lpfA segment-F	ACATATGCACCATCATCATCATCATGCTGAATCTGGTGACGGCAC
lpfA segment-R	AAGCTTGTCGACGGAGCTCGAATTCAACGTGAGCGGTTCATGATT
safA segment-F	ACATATGCACCATCATCATCATCATGGCTCGCTCGTGCCGAATAC
safA segment-R	AAGCTTGTCGACGGAGCTCGAATTCTTAAGCTTGATACTCCGCTA
stdA segment-F	ACATATGCACCATCATCATCATCATGTCGATCCACCCACAGCAGG
stdA segment-R	AAGCTTGTCGACGGAGCTCGAATTCTCACAGGTATTTCAGGGTGT
sthA segment-F	ACATATGCACCATCATCATCATCATCAGAACACGATTACGTTCAA
sthA segment-R	AAGCTTGTCGACGGAGCTCGAATTCTTACTGATACGAAACGGTAT

Procedures for Four Mutants of Wild-type Strain R51

The two-plasmid-based (pTargetF and pCas) CRISPR/Cas9 mediated genome-editing system was employed to created three individual mutants and one triple-deficient strain [28]. The primers are listed in the **Table S2**. Take the *citAB* mutant for example of individual mutant, the sgRNA (20-bp region) was designed through the online web server (http://chopchop.cbu.uib.no). The vector pTarget F was used as the template. The primers *cit*-sgRNA-F/sgRNA-R and sgRNA vector-F/R were used to amplify the small fragment with sgRNA sequence and the corresponding large vector fragment, respectively. The recombinase was used to ligate the two fragments to obtain the pTargetF (sgRNA), which chemically transformed into *E.* coli TG1 competent cell and spread onto the LB plate with spectinomycin. The single colonies were picked up and identified by PCR. Then the genome of the strain was used as the template. The primers *cit*-Donor DNA-Xf-F/R and cit-Donor DNA-Xr-F/R were used to amplify the Xf and Xr fragments, respectively. The donor DNA (Xf and Xr) was obtained by overlap PCR. The vector pTarget T (containing sgRNA and Donor DNA) was obtained by the same method as above. Prepare the R51 competent cells. L-arabinose-induced plasmid pCas was chemically transformed into R51. The single colonies growing on the LB plate with kanamycin were pick up and identified by PCR. The vector pTarget T was transformed into R51 harbouring the plasmid pCas and spread onto the LB plate with kanamycin and spectinomycin. After about three days, the single colony grown on the plate was confirmed by PCR whether the target fragment was deleted. The target colony harboring both pCas and pTargetT was inoculated into LB medium containing kanamycin and IPTG (isopropyl-D-thiogalactopyranoside, 0.5 mM) to cure pTargetT. After curing the pTargetT, the colony was stored at -80 °C for construction of double deletion mutant and used to cure the plasmid pCas. Because pCas is a temperature-sensitive plasmid which cannot maintain at 42 °C, pCas was cured by growing the colony overnight at 42°C nonselectively to obtain clean and traceless single deletion strain. The Δ*dcuSR* and Δ*dpiBA* mutants were also obtained in the same protocol. The triple-deficient strain was created based on a double strain and the methods were the same as above.
